# Motives and barriers to initiation and sustained exercise adherence in a fitness club setting—A one‐year follow‐up study

**DOI:** 10.1111/sms.13736

**Published:** 2020-06-15

**Authors:** Christina Gjestvang, Frank Abrahamsen, Trine Stensrud, Lene A. H. Haakstad

**Affiliations:** ^1^ Department of Sports Medicine Norwegian School of Sports Sciences Oslo Norway; ^2^ Department of Coaching and Psychology Norwegian School of Sports Sciences Oslo Norway

**Keywords:** adherence, barriers, exercise, fitness club members, fitness clubs, motives

## Abstract

No prospective studies have investigated motives and barriers to exercise in new untrained fitness club members. The aims of the present prospective longitudinal study were to (a) examine proportions reporting regular exercise, non‐regular exercise, and exercise dropout; (b) identify motives and barriers to exercise; and (c) compare motives between regular and non‐regular exercisers the first year of fitness club membership. New members (n = 250) were followed for 1 year. A questionnaire including demographics, exercise frequency, motives (EMI‐2), and barriers (18 common reported barriers) was used, and 184 answered at four time points (onset, and after 3, 6, and 12 months). Participants were categorized into *regular exercise:* ≥2 sessions/wk or *non‐regular exercise:* ≤1 session/wk, exercise relapse, or dropout. At 3, 6, and 12 months, 63.4%, 59.6%, and 57.2% exercised regularly, whereas 20.1%, 21.1%, and 28.3%, dropped out, respectively. Throughout the follow‐up, 37% reported regular exercise. At all time points, motives regarding positive health and strength/endurance were rated highest on a six‐point scale. Exercise dropouts rated priority as the greatest barrier. Regular exercisers rated the motives enjoyment (such as “I enjoy the feeling of exerting myself”) and challenge (such as “To give me goals to work towards”) higher than non‐regular exercisers (*P* = ≤.05). In conclusion, less than half exercised regularly, and most members were motivated by factors such as positive health and physical fitness the first year of fitness club membership. Higher levels of the motives enjoyment and challenge were associated with regular exercise.

## 1 INTRODUCTION

Regular exercise is associated with a wide range of well‐known health benefits such as prevention of cardiovascular diseases and type 2 diabetes.^1^ However, it appears to be challenging to adhere to regular exercise, and it is demonstrated that around 50% relapse to physical inactivity or a less active status the first months after initiation of exercise.^2^, ^3^ Hence, it is important to motivate physically inactive individuals to begin with exercise, and to encourage exercise adherents to maintain exercise.^4^ Many psychological factors influence exercise adherence, for instance, perceived motives and barriers.^5^

Perceived motives and barriers are key factors that influence initiation and regular exercise adherence.^6^ Self‐determination theory (SDT)^5^ is a contemporary theory that has been applied in the exercise domain and delineates how motives influence behavior. SDT suggests that motivation lies along a continuum of different degrees of autonomy. Behavior is considered to be freely initiated when the individual chooses to achieve a particular motive for autonomous rather than controlled reasons.^5^ Motives are autonomous when they are undertaken because of the value in itself, or because the motives are an important part of an individual's identity and controlled when they are initiated due to a sense of external or internal pressure.^5^, ^6^ Autonomously motivated individuals may exercise for the inherent enjoyment, because their motives are to achieve valued outcomes or are an important part of their identity. Individuals have controlled motivation when they are achieving motives to satisfy the wishes of some external pressure (eg, family/physician) or internal pressure (eg, sense of guilt).^5^, ^6^ Consistent with SDT, it is shown that more autonomous motives rather than more controlled motives are associated with regular exercise behavior.^7^

Further, the perception of barriers may inhibit an individual's exercise behavior, because barriers are significant predictors of physical activity.^8^ Perceived barriers encompass internal (eg, “I do not have time and energy”) and external components (eg, practical or environmental causes).^8^ Internal barriers are related to personal aspects, unlike external barriers, that refers to, for example, infrastructure in communities and practical barriers. The interaction of perceived barriers may particularly hinder leisure‐time exercise.^8^ There is consensus in the literature that access to exercise facilities (environmental factors), enjoyment (intrinsic motives), and fulfillment of goals positively influence exercise adherence, whereas lack of time, social support, and energy (internal barriers) inhibit exercise adherence.^9^, ^10^, ^11^ It is important to investigate motives and barriers in the context and setting where such activities take place, and it is unclear how motives and barriers to exercise in a fitness club setting are different from motives and barriers to exercising elsewhere.

The number of fitness clubs has increased significantly in recent decades.^12^ Worldwide, the fitness club industry has about 183 million members and more than 210 000 clubs; hence, it is one of the most popular settings for exercise.^12^ Fitness clubs are located where people live, work, and travel; have flexible opening hours; and offer childcare, in addition to a wide range of exercise opportunities.^12^ Fitness clubs may suit our “modern” lifestyle, which seldom offers occupational or commuting physical activity.^13^ Despite the increasing popularity of fitness clubs, several studies have found exercise dropout rates between 40% and 65% the first 5‐8 months after individuals join a fitness club.^14^, ^15^ Studies have also shown a trend (49%‐71%) of exercise relapse ^14^, ^16^, ^17^—an individual maintaining exercise for a period, then dropout for a short‐term, and then return to previous exercise behavior.^18^ Based on these numbers, it is important to investigate why some individuals adhere to regular exercise, while others relapse or dropout.

To our knowledge, only seven studies have reported on motives or barriers in a fitness club setting.^17^, ^19^, ^20^, ^21^, ^22^, ^23^, ^24^ However, six of these studies did not recruit untrained new fitness club members,^17^, ^19^, ^20^, ^21^, ^22^, ^24^ six were cross‐sectional,^17^, ^19^, ^20^, ^22^, ^23^, ^24^ and four are more than 10 years old.^17^, ^19^, ^20^, ^23^ In the fitness club industry, exercise has often been promoted in relation to external outcomes, such as appearance.^25^ However, the fitness club industry has evolved substantially over the last decade.^25^ To make gym culture more accessible to everyone, fitness clubs have shifted toward a more body‐positive, health‐related focus.^25^ To date, the “typical fitness club” offers exercise options that should make you feel good, instead of “looking good.” However, we do not know whether this shift also has influenced the motives of those who choose to join a gym, especially new recreational exercisers. Individuals' motives to initiate exercise may also differ from the motives that lead to sustained exercise adherence. Hence, the present study bridges this gap by identifying motives and barriers that are contributing to regular use of the gym, not only the first weeks but also months after joining a fitness club.

This study aimed to examine the proportions reporting regular exercise, non‐regular exercise, and exercise dropout, as well as to identify perceived motives and barriers to exercise throughout the first year of fitness club membership. Thirdly, we wanted to compare motives between those who reported regular exercise with those who did not (irregular exercise or exercise dropout) at 3, 6, and 12 months.

## 2 MATERIALS AND METHODS

For the present study, we used data from the research project *Fitness clubs—a venue for public health?*, a 1‐year follow‐up study conducted in Oslo (Norway) from October 2015 to October 2018.^26^, ^27^ The main aim of the project was to investigate factors associated with exercise adherence and dropout in a group of new beginner exercisers in a fitness club setting.^26^, ^27^ Hence, motives and barriers were one of the project's primary outcomes. All new members from 25 multipurpose gyms (resistance and cardio‐exercise rooms, and group exercise classes) in one fitness club chain (mid‐ to high membership fees) were invited to take part in the study by e‐mail invitation. In total, 676 individuals wanted to participate in the study, of whom 148 did not respond after the first e‐mail. Enrollment was limited to adults (≥18 years), <4 weeks membership, classified as non‐exercising (exercising <60 min/wk at moderate or vigorous intensity or brisk walking <150 min/wk, in the last 6 months),^28^ and healthy (no disease or illness considered to hinder physical activity, eg, severe heart disease, hypertension, or lung diseases such as asthma). We excluded 278 who did not meet the eligibility criteria (physically active n = 270, disease/illness n = 8). Hence, 250 fitness club members were included. More details of the research project are published elsewhere.^26^, ^27^

### 2.1 Ethical approval

The Norwegian Social Science Data Service provided approval for this study (NSD 44135). The project was reviewed by the Regional Committee for Medical and Health Research Ethics (REK 2015/1443 A) that concluded that according to the Act on Medical and Health Research (the Health Research Act 2008), the study did not require extensive review. All participants signed informed consent for participation in the study, following the Helsinki Declaration.

### 2.2 Outcome measures

A standardized electronic questionnaire was used to obtain demographic information, exercise involvement, perceived motives, and barriers. At all time points (at onset, 3, 6, and 12 months of fitness club membership), the questionnaire took approximately 25 minutes to complete and was answered by 250, 224, 213, and 187 participants, respectively. A total of 184 participants answered at all four time points. Losses to follow‐up included life situation (n = 16), injury/disease (n = 6), relocation (n = 1), and unknown reasons (n = 43).

The specific questionnaire section concerning motives for exercise was based on the validated questionnaire *Exercise Motivations Inventory‐2 (EMI‐2)* ^29^ and translated into Norwegian by three members of the research group. Due to a comprehensive questionnaire in the current research project and 16 statements not considered relevant in a fitness club setting (such as “Because I like trying to win in physical activities” and “Because I enjoy physical competition”), we chose 35 out of 51 statements from the original EMI‐2. The EMI‐2 consists of 14 different subscales that can be considered as extrinsic or intrinsic motives, and each subscale includes one to four statements.^29^ The participants were requested to rate the significance of each statement as a personal motive for exercise on a six‐point scale, ranging from 0 (not true for me) to 5 (very true for me). Further, a sum score for each subscale was calculated. The participants could also tick “I do not want to answer.”

Assessment of barriers to exercise was based on a former investigation in Norway in which an adult population (n = 12 504) reported on perceived barriers to physical activity,^30^ and a pilot testing completed among four volunteers and four research group members. We included all barriers and subscales used in that study, and added four barriers suggested to be an issue for members in a fitness club: “I do not know how to exercise,” “I am embarrassed for others to see me exercise,” “I am afraid to do the exercises wrong,” and “I am afraid of injuries.” Based on the initial investigation, we also categorized the perceived barriers into four subscales: priority, practical, health‐related, and affective‐cognitive.^30^ Each subscale included two to nine statements, and the participants were asked to rate the significance of each statement on a three‐point scale, ranging from 1 (not important to me) to 3 (very important to me).^30^ Then, a sum score for each subscale was calculated. Perceived barriers to exercise were answered by all participants at onset of fitness club membership (n = 184). In the electronic questionnaire, only those who reported exercise dropout at 3 (=43), 6 (n = 53), and 12 months (n = 65) were forwarded to statements regarding barriers. Overview of subscales and sample statements on motives and barriers to exercise is presented in Table 1.

**Table 1:** Subscales and sample statements of perceived motives and barriers to exercise

Subscales	Number of items	Sample statements
Motives^a^		
Stress Management	3	To help manage stress
Revitalization	2	To recharge my batteries
Enjoyment	4	Because I enjoy the feeling of exerting myself
Challenge	3	To give me goals to work towards
Social Recognition	2	To gain recognition for my accomplishments
Affiliation	4	To spend time with friends
Competition	1	Because I enjoy competing
Health Pressures	3	Because my doctor advised me to exercise
Ill‐Health Avoidance	3	To prevent health problems
Positive Health	3	To have a healthy body
Weight Management	4	To lose weight
Appearance	4	To look more attractive
Strength and Endurance	3	To increase my endurance
Mobility	2	To stay/become flexible
Barriers^b^		
Priority	2	I do not have time and energy
Practical	4	I lack transport
Health‐related	3	Health problems hinder me
Affective‐cognitive	9	I do not like to be physically active

^a^ Answered by all participants at onset, and after 3, 6, and 12 mo (n = 184).

^b^ Answered by all participants at onset (n = 184) and participants reporting exercise dropout at 3 (n = 43), 6 (n = 53), and 12 mo (n = 65).

At the 3‐, 6‐, and 12‐month follow‐ups, the participants also reported on exercise involvement. The questions and response options were as follows: (a) “Are you still a fitness club member?”: “yes” or “no”; (b) “Have you been exercising regularly?”: “yes” or “no”; (c) “How often have you exercised per week on average at the fitness club?”: “number of sessions”; and (d) “How often have you exercised per week on average outside the fitness club?”: “number of sessions.” In the analysis, questions 3 and 4 were amassed to the total number of sessions/wk. We asked the participants to report exercise involvement over only the last 4 weeks, due to potential recall bias associated with the use of self‐report.^31^

In line with definitions suggested by Hawley‐Hague,^4^ participants self‐reported exercise involvement across all three time points were divided into *regular exercise* (n = 68), reporting ≥2 exercise sessions/wk and *non‐regular exercise* (n = 116), reporting ≤1 exercise session/wk, exercise relapse (eg, reported exercise at 3 and 12 months, and no exercise at 6 months), or exercise dropout (reported no exercise during the follow‐up period). Regular exercise was based on that ≥2 exercise sessions/wk is suggested to improve factors such as physical fitness and health.^28^

### 2.3 Statistical analysis

Sample size considerations for the present study were based on studies assessing motives to exercise (EMI‐2) among adults,^32^, ^33^ as well as what the research group hypothesized to be relevant changes in scores on motives for new members joining a fitness club. All equations were based on detecting a 10% change in every single motive statement using univariate and bivariate analyses $N={\frac{\sigma^{2}\left(z_{1-\beta }+z_{1-\alpha /2}\right)}{{\left(\mu_{0}-\mu_{1}\right)}^{2}}}^{2}$ . With a power of 80% at the 0.05 level, we would be able to detect a 10% change in, for example, the subscales “Enjoyment” and “Challenge” with 137 and 154 participants, respectively. To allow adjustment of other factors and losses to follow‐up, 30% more participants were needed.^34^ We aimed to recruit all new fitness club members who fulfilled the eligibility criteria between October 2015 and October 2017.

The data were analyzed using SPSS Statistical Software (version 24.0 for Windows). Results are presented as frequencies (n) and percentages or means with standard deviations (SD), as well as 95% confidence intervals (CIs) and effect sizes (d). To investigate differences between regular and non‐regular exercisers in background variables (age, body weight, gender, body mass index, educational level, total household income, cohabitation, and occupation) at onset, an independent *t* test or chi‐square test was used as appropriate. To examine changes in motives and barriers between onset, 3, 6, and 12 months and differences between regular and non‐regular exercisers, a one‐way repeated‐measures ANOVA with Bonferroni correction and an independent *t* test were used, respectively. A *P*‐value ≤ .05 was considered to indicate statistical significance, with a cut‐off value of *P* = ≤.012 for the Bonferroni correction. Effect sizes were interpreted as small (0.20), medium (0.50), and large (0.80).^35^ To compare motives between those who reported regular exercise with those who did not, only participants who completed the questionnaire at all time points were included in the analysis (n = 184).

## 3 RESULTS

A total of 79.9% were still fitness club members at 12‐month follow‐up. Among all participants, at 3, 6, and 12 months, 63.4%, 59.6%, and 57.2% reported regular exercise, whereas 20.1%, 21.1%, and 28.3% had dropped out, respectively. Of 184 participants that completed the full study (who answered the questionnaire at all time points), 37.0% were classified as regular exercisers throughout the first year of fitness club membership, with an average of 3.88 (SD 1.66) exercise sessions/wk. Of those classified as non‐regular exercisers (63.0%), exercise dropout was reported by 38.8%, 48.3%, and 56.0% at 3, 6, and 12 months, respectively. Sixteen participants (13.8%) did not start exercising at all.

Nearly half of early exercise dropouts reported exercise at 6 (51.2%) and 12 months (39.1%), and 45.6% of exercise dropouts at 6 months exercised again at 12 months. Of those relapsing, 61.4% reported exercise dropout only once. Further, of those exercising ≤1 session/wk at 3 months, 46.7% and 53.3% reported exercise ≥2 sessions/wk at 6 and 12 months, respectively. At 12 months, 60.0% of those reporting ≤1 exercise session/wk at 6 months exercised ≥2 sessions/wk.

Concerning background variables at onset, a larger proportion of those classified as regular exercisers throughout all three follow‐ups were men, overweight/obese (BMI ≥ 25), older, and employed outside the home, compared with non‐regular exercisers (63.0%) (Table 2). The two groups were well‐balanced in household income, education, and cohabitation. The principal reasons for membership dropout, health variables, physical fitness, and physical activity level are described elsewhere.^26^, ^27^

**Table 2:** Background characteristics of participants divided into regular exercisers (n = 68) and non‐regular exercise (n = 116) throughout all three follow‐ups

Variable	Regular exercisers	Non‐regular exercise	*P*
	Mean ± SD	Mean ± SD	
Age (y)	39.5 ± 12.5	35.5 ± 10.7	.028
Body weight (kg)	82.8 ± 14.7	77.3 ± 15.0	.017
	n (%)	n (%)	
Gender (men)	43 (63.2)	51 (44.0)	.018
BMI (kg/m^2^) ≥25 (overweight or obese)	43 (63.2)	53 (45.7)	.032
High educational level (≥4 y of higher education)	30 (44.1)	47 (40.5)	.747
High household income (>100 000 US dollar per year)	29 (42.7)	39 (33.6)	.286
Spouse/partner	42 (61.8)	73 (63.0)	.875
Have children	19 (27.9)	38 (32.8)	.605
Employed outside the home	57 (83.8)	78 (67.2)	.022

Abbreviation: Body mass index.

### 3.1 Perceived motives and barriers throughout the first year of fitness club membership

At all follow‐ups, the motives positive health (4.37‐4.51), increase in strength/endurance (3.76‐4.00), and mobility (3.63‐3.92) were rated highest on a six‐point scale (Table 3). Throughout the follow‐up, we found an increase in six subscales of motives: appearance (d = 0.13), enjoyment (d = 0.13), challenge (d = 0.06), stress management (d = 0.10), health pressures (d = 0.19), and social recognition (d = 0.11), with 0.26‐0.52 higher scores at 3, 6, and 12 months, compared with onset. However, despite an increase, three subscales (enjoyment, challenge, and stress management) had scores below the midpoint of the scale (from 0 to 5) (Table 3). We also found a decrease in the subscales strength and endurance from midway (in mean 0.22‐0.24 lower scores) to 12‐month follow‐ups. The score at 12 months was also lower compared with onset (Table 3).

**Table 3:** Subscales of perceived motives to and barriers for exercise at onset, and after 3, 6, and 12 mo

Subscales (0‐5)	Onset	3 mo	6 mo	12 mo		
	(n = 184)	(n = 184)	(n = 184)	(n = 184)		
	Mean ± SD (95% CI)	Mean ± SD (95% CI)	Mean ± SD (95% CI)	Mean ± SD (95% CI)	Cohen`s d	*P*
Positive Health	4.47 ± 0.82 (4.34, 4.58)	4.51 ± 0.82 (4.38, 4.62)	4.41 ± 0.80 (4.30, 4.53)	4.37 ± 0.82 (4.25, 4.49)	0.02	.217
Strength and Endurance	3.84 ± 1.01 (3.69, 3.99)	4.00 ± 1.20 (3.85, 4.13)**	3.98 ± 1.12 (3.81, 4.15)**	3.76 ± 1.10 (3.58, 3.93)	0.06	.012
Mobility	3.63 ± 1.39 (3.42, 3.83)	3.92 ± 1.24 (3.75, 4.11) *	3.83 ± 1.39 (3.61, 4.04)	3.74 ± 1.36 (3.55, 3.92)	0.04	.028
Ill‐Health Avoidance	3.56 ± 1.18 (3.38, 3.73)	3.82 ± 0.91 (3.68, 3.95)*	3.76 ± 1.05 (3.60, 3.90)	3.69 ± 1.12 (3.52, 3.86)	0.04	.028
Weight Management	3.18 ± 1.61 (2.95, 3.41)	3.39 ± 1.44 (3.18, 3.60)	3.54 ± 1.46 (3.33, 3.75)*	3.46 ± 1.48 (3.24, 3.67)*	0.08	.001
Appearance	2.98 ± 1.41 (2.76, 3.18)	3.30 ± 1.40 (3.09, 3.50)*	3.37 ± 1.38 (3.17, 3.57)*	3.25 ± 1.37 (3.05, 3.45)*	0.13	<.001
Revitalization	2.97 ± 1.28 (2.78, 3.16)	3.10 ± 1.30 (2.91, 3.28)	3.14 ± 1.27 (2.96, 3.33)	3.06 ± 1.32 (2.86, 3.25)	0.02	.187
Enjoyment	2.47 ± 1.40 (2.25, 2.66)	2.83 ± 1.42 (2.60, 3.03)*	2.87 ± 1.39 (2.66, 3.06)*	2.74 ± 1.47 (2.53, 2.95)*	0.13	<.001
Competition	1.11 ± 1.56 (0.90, 1.34)	1.11 ± 1.51 (0.90, 1.33)	1.18 ± 1.57 (0.97, 1.42)	1.29 ± 1.76 (1.05, 1.56)	0.01	.320
Challenge	1.91 ± 1.43 (1.72, 2.13)	2.20 ± 1.49 (1.99, 2.42)*	2.19 ± 1.59 (1.98, 2.43)*	2.25 ± 1.58 (2.02, 2.48)*	0.06	.006
Stress Management	1.94 ± 1.45 (1.73, 2.15)	2.20 ± 1.46 (2.00, 2.43)*	2.28 ± 1.58 (2.04, 2.52)*	2.36 ± 1.52 (2.13, 2.60)*	0.10	<.001
Health Pressures	1.11 ± 1.23 (0.94, 1.30)	1.63 ± 1.33 (1.44, 1.82)*	1.49 ± 1.45 (1.29, 1.70)*	1.55 ± 1.51 (1.33, 1.79)*	0.19	<.001
Social Recognition	0.64 ± 1.01 (0.51, 0.79)	0.90 ± 1.12 (0.73, 1.07)*	1.00 ± 1.27 (0.82, 1.18)*	0.99 ± 1.33 (0.81, 1.19)*	0.11	<.001
Affiliation	0.87 ± 1.05 (0.72, 1.04)	0.96 ± 1.16 (0.79, 1.14)	1.03 ± 1.29 (0.84, 1.23)	1.10 ± 1.34 (0.91, 1.30)	0.03	.107
Barrier subscales (1‐3)	(n = 184)	(n = 43)	(n = 53)	(n = 65)		
	Mean ± SD (95% CI)	Mean ± SD (95% CI)	Mean ± SD (95% CI)	Mean ± SD (95% CI)		*P* ^†^
Priority	2.03 ± 0.63 (1.71, 2.35)	2.17 ± 0.69 (1.78, 2.53)	2.32 ± 0.46 (2.07, 2.57)	2.17 ± 0.54 (1.89, 2.42)	0.32	.206
Practical	1.50 ± 0.51 (1.28, 1.82)	1.39 ± 0.41 (1.21, 1.62)	1.41 ± 0.36 (1.23, 1.60)	1.37 ± 0.37 (1.19, 1.58)	0.11	.714
Health‐related	1.52 ± 0.33 (1.35, 1.69)	1.64 ± 0.49 (1.38, 1.90)	1.57 ± 0.35 (1.38, 1.76)	1.45 ± 0.33 (1.30, 1.64)	0.24	.365
Affective‐cognitive	1.35 ± 0.30 (1.12, 1.57)	1.38 ± 0.32 (1.16, 1.62)	1.44 ± 0.25 (1.24, 1.61)	1.29 ± 0.31 (1.11, 1.55)	0.56	.424

^†^ Only participants who reported exercise dropout at all time points (n = 16) were included in the analysis,

* significantly different from onset, and

** significantly different from 12 mo.

At 3, 6, and 12 months, the internal barrier priority, on a three‐point scale (2.03‐2.32, d = 0.32), was rated as the most important among exercise dropouts. Otherwise, all other barrier subscales had scores around the midpoint of the scale (1‐3) and remained relatively unchanged throughout the 1‐year follow‐up (Table 3). The barrier statements suggested to be an issue for members in a fitness club (“I do not know how to exercise,” “I am embarrassed for others to see me exercise,” “I am afraid to do the exercises wrong,” and “I am afraid of injuries”) had mean scores below the midpoint of the scale (3 months: 1.37 ± 0.65, 6 months: 1.27 ± 0.57, 12 months: 1.38 ± 0.67).

We found no persistent gender differences in perceived motives or barriers throughout the first year of fitness club membership. For brevity, these are not included.

Regular exercisers rated the subscales enjoyment (mean diff. from 0.67 to 0.80, d = 0.06 to 0.09) and challenge (mean diff. from 0.50 to 0.69, d = 0.004 to 0.03) higher than non‐regular exercisers at all four measurements points (Table 4).

**Table 4:** Subscales of perceived motives to exercise at onset (all participants n = 184), and after 3, 6, and 12 mo between regular exercise (n = 68) and non‐regular exercise (n = 116)

Subscales (0‐5)	Onset	3 mo			6 mo			12 mo		
	All	Regular exercise	Non‐regular exercise	*P*	Regular exercise	Non‐regular exercise	*P*	Regular exercise	Non‐regular exercise	*P*
	Mean ± SD (95% CI)	Mean ± SD (95% CI)	Mean ± SD (95% CI)		Mean ± SD (95% CI)	Mean ± SD (95% CI)		Mean ± SD (95% CI)	Mean ± SD (95% CI)	
Positive Health	4.46 ± 0.82 (4.36, 4.56)	4.51 ± 0.95 (4.27, 4.73)	4.50 ± 0.74 (4.36, 4.63)	.964	4.36 ± 0.88 (4.15, 4.56)	4.43 ± 0.76 (4.29, 4.57)	.599	4.33 ± 0.88 (4.10, 4.54)	4.39 ± 0.80 (4.23, 4.54)	.645
Strength and Endurance	3.86 ± 1.08 (3.72, 3.99)	4.17 ± 0.80 (3.98, 4.37)	3.89 ± 1.13 (3.68, 4.10)	.050	4.13 ± 0.86 (3.93, 4.32)	3.91 ± 1.25 (3.68, 4.13)	.213	3.92 ± 1.05 (3.65, 4.16)	3.78 ± 1.19 (3.54, 3.97)	.397
Mobility	3.63 ± 1.45 (3.44, 3.82)	4.05 ± 1.22 (3.74, 4.34)	3.85 ± 1.25 (3.62, 4.06)	.278	4.08 ± 1.23 (3.78, 4.35)	3.68 ± 1.46 (3.41, 3.93)	.055	3.89 ± 1.12 (3.63, 4.16)	3.66 ± 1.47 (3.37, 3.92)	.275
Ill‐Health Avoidance	3.64 ± 1.18 (3.50, 3.79)	3.95 ± 0.86 (3.71, 4.15)	3.75 ± 0.94 (3.56, 3.91)	.143	3.95 ± 1.06 (3.69, 4.20)	3.64 ± 1.04 (3.47, 3.83)	.057	3.76 ± 1.07 (3.47, 4.00)	3.66 ± 1.15 (3.45, 3.85)	.540
Weight Management	3.15 ± 1.58 (2.95, 3.35)	3.45 ± 1.41 (3.13, 3.76)	3.37 ± 1.47 (3.08, 3.61)	.698	3.67 ± 1.43 (3.31, 3.99)	3.48 ± 1.48 (3.23, 3.75)	.384	3.68 ± 1.33 (3.34, 4.00)	3.35 ± 1.56 (3.07, 3.62)	.125
Revitalization	3.02 ± 1.33 (2.85, 3.18)	3.27 ± 1.18 (3.01, 3.53)	3.00 ± 1.36 (2.76, 3.23)	.164	3.52 ± 1.16 (3.24, 3.80)	2.93 ± 1.29 (2.70, 3.16)	.002	3.36 ± 1.19 (3.06, 3.64)	2.89 ± 1.37 (2.64, 3.13)	.015
Appearance	3.07 ± 1.34 (2.90, 3.25)	3.29 ± 1.39 (2.97, 3.58)	3.31 ± 1.42 (3.03, 3.56)	.940	3.48 ± 1.39 (3.14, 3.80)	3.23 ± 1.38 (2.95, 3.53)	.561	3.19 ± 1.35 (2.87, 3.51)	3.29 ± 1.39 (3.04, 3.56)	.609
Enjoyment	2.57 ± 1.44 (2.39, 2.75)	3.28 ± 1.19 (3.00, 3.56)	2.56 ± 1.47 (2.30, 2.83)	.001	3.42 ± 1.22 (3.12, 3.71)	2.55 ± 1.39 (2.31, 2.80)	<.001	3.21 ± 1.26 (2.88, 3.51)	2.47 ± 1.52 (2.20, 2.74)	.001
Challenge	2.05 ± 1.52 (1.87, 2.23)	2.65 ± 1.45 (2.31, 2.98)	1.93 ± 1.45 (1.67, 2.19)	.001	2.78 ± 1.53 (2.41, 3.13)	1.85 ± 1.54 (1.59, 2.12)	<.001	2.67 ± 1.39 (2.34, 3.01)	1.99 ± 1.64 (1.68, 2.31)	.004
Stress Management	2.02 ± 1.51 (1.84, 2.21)	2.34 ± 1.47 (2.00, 2.69)	2.12 ± 1.45 (1.87, 2.38)	.325	2.63 ± 1.57 (2.27, 3.02)	2.07 ± 1.55 (1.79, 2.35)	.020	2.60 ± 1.44 (2.27, 2.94)	2.22 ± 1.55 (1.95, 2.50)	.100
Competition	1.19 ± 1.55 (0.99, 1.38)	1.19 ± 1.45 (0.84, 1.53)	1.06 ± 1.55 (0.79, 1.34)	.593	1.41 ± 1.44 (1.07, 1.90)	1.01 ± 1.46 (0.73, 1.28)	.059	1.17 ± 1.64 (0.80, 1.61)	1.34 ± 1.83 (1.01, 1.66)	.515
Health Pressures	1.12 ± 1.22 (0.98, 1.27)	1.82 ± 1.45 (1.50, 2.17)	1.52 ± 1.24 (1.29, 1.74)	.153	1.61 ± 1.48 (1.24, 1.96)	1.41 ± 1.44 (1.16, 1.66)	.359	1.59 ± 1.45 (1.24, 1.95)	1.53 ± 1.55 (1.24, 1.82)	.787
Social Recognition	0.72 ± 1.05 (0.59, 0.85)	0.91 ± 1.18 (0.63, 1.19)	0.90 ± 1.09 (0.70, 1.10)	.951	1.16 ± 1.39 (0.85, 1.51)	0.90 ± 1.19 (0.70, 1.13)	.196	0.83 ± 1.09 (0.59, 1.11)	1.09 ± 1.45 (0.85, 1.36)	.208
Affiliation	0.94 ± 1.05 (0.80, 1.07)	1.00 ± 1.15 (0.72, 1.28)	0.93 ± 1.16 (0.72, 1.15)	.681	1.31 ± 1.35 (0.98, 1.64)	0.87 ± 1.24 (0.66, 1.11)	.031	1.07 ± 1.11 (0.82, 1.35)	1.11 ± 1.47 (0.85, 1.39)	.878

Note

*P*‐value shows differences between regular exercise and non‐regular exercise.

## 4 DISCUSSION

The main findings were that few (37%) maintained regular exercise throughout the first year of fitness club membership. At 3, 6, and 12 months, regular exercisers rated motives such as enjoyment and challenge higher than non‐regular exercisers. However, the differences in means and magnitude of the effect sizes for the motives enjoyment and challenge were small. Our results suggest that those exercising regularly are more likely to report that they exercise for the inherent enjoyment.

Consistent with other studies among fitness club members, our study also demonstrates low exercise adherence and an increase in exercise dropout throughout the initial year of fitness club membership.^14^, ^15^ However, only 13.8% of non‐regular exercisers reported sustained exercise dropout at all time points. Hence, the majority relapsed, a common phenomenon at fitness clubs.^17^ In agreement with the literature,^20^, ^22^, ^24^ we also found that the most common barrier was priority (such as finding time to exercise). Therefore, it may be essential that fitness club staff promotes practical methods toward members on how to exercise regularly, such as planning (creating time for exercise in one's schedule), and how to incorporate exercise into everyday life.

In our study, motives with external outcomes such as positive health and an increase in physical fitness were reported as the main motives for exercise, consistent with two studies among fitness club members.^23^, ^24^ Other authors investigating motives for exercise among individuals in different activity settings have revealed that fitness club members are more likely to report motives such as appearance than motives such as social factors and enjoyment, compared with individuals exercising at sports clubs or in public spaces.^36^, ^37^ SDT proposes that individuals may engage in exercise to obtain outcomes separate from the behavior itself, such as physical fitness and appearance‐related goals, and individuals may value their exercise goals differently.^5^, ^6^, ^38^ For instance, to achieve positive changes in physical fitness, an untrained individual must exercise >1 session/wk over a specific period (eg, 12 weeks).^28^ If progress is lacking and the individual's motive is undertaken for controlled reasons (by, eg, external pressure), this may contribute to exercise relapse or dropout. In contrast, more autonomous motives (eg, the individual value higher levels of physical fitness) may predict sustained exercise adherence.^7^ Hence, it may be the reason why an individual has a particular exercise motive or goal that results in exercise behavior.^5^ An individual may exercise to improve physical fitness (an external outcome) to satisfy an external demand such as a doctor (controlled), to avoid feelings of, for example, guilt (controlled), because the individual values physical fitness (autonomous), or consistent with his or her ambitions in life (autonomous).^5^ Therefore, all motives have an autonomous or controlled foundation, and it is shown that the strongest predictor of exercise maintenance is whether the individual personally values the outcome (eg, higher physical fitness).^11^ Fitness club staff may benefit by paying attention to the members’ exercise goals and the motivation attributed to the goals, due to the relationship between goals and motivation.^38^ If the members’ motives are commenced for controlled reasons, it is essential to guide the member to create more autonomous motives. In our study, both non‐regular exercisers and regular exercisers had high scores on motives related to external outcomes (such as strength/endurance), and we may speculate whether regular exercisers were more autonomously motivated than the non‐regular exercisers. However, in the present study, we did measure exercise motives only. Another explanation that the participants in our study had high scores on the motives positive health, increase in physical fitness, and mobility may be that individuals motivated by external outcomes join a fitness club because it appears to be an activity setting that fits their goals.^25^

Several studies among fitness club members^20^, ^22^, ^24^ and the general population^30^, ^39^ demonstrate that lack of time and motivation are the most common barriers that inhibit exercise adherence. This is in line with our findings, where priority (lack of time/energy or valuing other leisure‐time activities) was perceived as the most important barrier. Despite paying monthly fees and despite access to exercise equipments and group exercise classes, 23.0%‐35.0% of our participants dropped out once or more during the follow‐up period. As most fitness clubs are conveniently located and offer practical solutions for exercise attendance (such as intense group exercise classes of 30 minutes and childcare), “lack of time” is both a barrier and perhaps an excuse. Further, most barriers were rated below or around midpoint on the scale by exercise dropouts, which can be seen as non‐limiting barriers. However, individuals cope differently with barriers, so to what extent a barrier is a limitation to exercise is suggested to be not automatic.^40^ It has also been proposed that the total number of perceived barriers is likely to be more important, because it may be easier to overcome one or a few barriers rather than many.^30^ It may be essential that fitness clubs may implement a tutorial talk for all new members, aiming to get an overview of possible barriers and how to overcome these barriers (such as low priority).^40^

### 4.1 Strengths and limitations

Collecting data as it happens in a real‐life natural context, a low dropout rate, and the use of a prospective longitudinal design with 12 months of follow‐up are considered strong aspects of the present study. Previous studies reporting on motives or barriers in a fitness club setting are cross‐sectional, and several were published more than 10 years ago. The present study had several follow‐ups throughout the first year of fitness club membership, allowing us to investigate changes in motives and barriers. Another strength was the use of an electronic questionnaire based on validated questions^29^ and a previous investigation in Norway.^30^ Electronic questionnaires are cost‐efficient and gather responses quickly. Further, we recruited from 25 fitness clubs, and the sample (untrained new fitness club members) is a study population of which there is limited knowledge. Sample size considerations estimated that fewer participants were needed than the number who participated. Also, subgroup analysis comparing regular exercisers with non‐regular exercisers allowed us to investigate the influence of intrinsic and extrinsic motives on exercise adherence.

Limitations were that data were obtained from only one fitness club chain. Recruitment of other gyms such as fitness‐only (low‐cost membership) and CrossFit gyms might have yielded different results. Confounding factors such as gender and age may also be present because we did not adjust for background variables. Losses to follow‐ups may also introduce selection bias; hence, the results should be viewed with caution. However, a comparative analysis of demographic data from study dropouts (n = 66, 26.4%) and current participants at 12 months indicated no differences in age, gender, educational level, total household income, or BMI. In the current study, another limitation was that exercise attendance was measured by self‐report, with no objective data of attendance at the fitness club. It is well known that individuals tend to overestimate the number of exercise sessions because of social desirability, and therefore, the measure may be imprecise.^31^ In addition, we defined two sessions/wk as regular exercise attendance, and this definition does not reflect if the participants met the current physical activity recommendations. Yet, with respect to exercise intensity, it is still possible to meet the physical activity recommendations by two exercise sessions/wk. However, we did not measure exercise intensity in the present study. Further, another limitation with using an electronic questionnaire is the absence of an interviewer or someone present to help interpret questions; also, an electronic questionnaire may not be suitable for asking open‐ended questions. We also considered comparing motives between regular exercisers, those with exercise relapse and exercise dropouts. Regrettably, our sample size was not large enough to statistically compare more than two groups. Another limitation is that only those reporting exercise dropout answered statements regarding barriers at 3, 6, and 12 months. Hence, we could not conduct a longitudinal analysis of barriers in all participants. Finally, our quantitative design may not be robust enough to explain complex aspects such as motives and barriers to exercise. Hence, there is a need for future qualitative studies investigating this in more depth.

## 5 PERSPECTIVES

After 1‐year follow‐up, more than half in our study were classified as non‐regular exercisers, despite being a gym member. Fitness club staff and specifically the instructors are in a unique position to influence members’ attitudes and exercise behavior. Aiming to increase the proportion that is adhering to regular exercise, it should be highlighted to fitness club staff that knowledge of SDT and how to translate theoretical principles into “real‐life” practice may be important for members’ exercise participation. An instructor with knowledge of the relationship between motives and behavior may know how to get the members aware of why they have a particular exercise motive and guide them to create more autonomous motives. Fitness club employees should also implement practical methods that seek to prevent barriers, through the understanding of behavior and the underlying mechanisms.^5^

## 6 CONCLUSION

Less than half (37.0%) of the participants reported regular exercise adherence throughout the first year of fitness club membership. Most members were motivated for exercise by factors such as positive health, increase in physical fitness, and mobility, and the most common barrier to exercise adherence was priority (such as lack of time). Regular exercisers rated the motives enjoyment and challenge as more important than non‐regular exercisers, however, the differences in means and magnitude of the effect sizes were very small.

## CONFLICTS OF INTEREST

There are no conflicts of interest, including financial, consultant, institutional, and other relationships that might lead to bias or a conflict of interest. The results of the study are presented clearly, honestly, and without fabrication, falsification, or inappropriate data manipulation.
